# Kinetics of Humoral Immunodeficiency With Bispecific Antibody Therapy in Relapsed Refractory Multiple Myeloma

**DOI:** 10.1001/jamanetworkopen.2022.38961

**Published:** 2022-10-28

**Authors:** Lindsay R. Hammons, Aniko Szabo, Abhishek Janardan, Binod Dhakal, Saurabh Chhabra, Anita D’Souza, Meera Mohan

**Affiliations:** 1 Division of Hematology and Oncology, Medical College of Wisconsin, Milwaukee

## Abstract

This case series describes the kinetics of humoral deficiency in patients with relapsed refractory multiple myeloma treated with bispecific antibodies, the infectious complications, and response to COVID-19 immunization.

## Introduction

Bispecific antibodies (bsAb) targeting novel antigens are a promising class of therapeutics for relapsed refractory multiple myeloma (RRMM).^1,2^ Although hypogammaglobulinemia is expected due to plasma cell depletion,^3,4,5^ little is known regarding the degree of humoral immunodeficiency and infectious complications with bsAb. We report on the kinetics of humoral deficiency among patients with RRMM treated with bsAb, the infectious complications, and response to COVID-19 immunization.

## Methods

In this case series, we included patients with RRMM treated in early phase 1 and 2 clinical trials of bsAb at our institution between January 1, 2019, and December 31, 2021. Patients self-reported their sex and race and ethnicity; the latter data were collected due to the high incidence of multiple myeloma among racial and ethnic minority groups. Serial immunoglobulin levels and infections confirmed by clinical, imaging, microbiologic, or histopathologic evidence were captured retrospectively from the start of therapy until last follow-up or 3 months after study exit. Cytokine release syndrome and immune effector cell–associated neurotoxicity syndrome were graded per American Society for Transplantation and Cellular Therapy criteria,^6^ and antibiotic prophylaxis was prescribed per current institutional guidelines. Statistical analysis details are provided in the eMethods in the Supplement.

The Medical College of Wisconsin Institutional Review Board approved this study and waived the need for informed consent owing to its retrospective design. This study followed the reporting guideline for case series.

## Results

Forty-two patients contributing 49 treatment periods (7 patients were counted twice) were included in our analysis (25 in men [51%] and 24 in women [49%]; median age, 67 [range, 44-84] years). The Table summarizes baseline characteristics. At a median follow-up of 9.5 (range, 0.9-28.6) months, bsAb therapy continued for 17 of 42 patients (40%). Serum IgG levels were 159 mg/dL at a median of 104 (range, 2-445) days from initiation of treatment. Serum IgA and IgM levels were both less than 5 mg/dL at a median of 23 (IQR, 2-379) and 21 (IQR, 2-296) days, respectively, from the start of therapy. Serum IgG levels were below the detectable range (<40 mg/dL) in 14 patients (28%) at some point during therapy; serum IgA and IgM levels were below the detectable range (<5 mg/dL) in 21 of 42 (50%) and 25 of 42 patients (60%), respectively (to convert immunoglobulin levels to g/L, multiply by 0.01). In 24 of 49 treatment periods (49%), intravenous immunoglobulin supplementation was received (Figure).

**Table.** Baseline Patient Characteristics

Characteristic	Values^a^
Sex	
Men	25 (51)
Women	24 (49)
Age, median (range), y	67 (44-84)
Race	
African American or Black	9 (18)
Asian	1 (2)
White	39 (80)
Use of up-front autologous stem cell transplant	47 (96)
Use of salvage autologous stem cell transplant	17 (35)
Revised International Staging System^b^	
I	5 (19)
II	14 (54)
III	7 (27)
International Staging System^c^	
I	16 (38)
II	11 (26)
III	15 (36)
Prior lines of therapy, median (range)	5 (2-11)
Baseline high-risk FISH^d^	16 (33)
Triple class refractory disease	47 (96)
Exposure to prior BCMA-targeting agent	7 (14)
Baseline severe hypogammaglobinemia (≤40 mg/dL)^e^	24 (49)
Baseline lymphopenia	12 (24)
BCMA-targeting bsAb	42 (86)
GPRC5D-targeting bsAb	7 (14)
CRS (all grades)	26 (53)
Use of tocilizumab	10 (20)
Use of systemic corticosteroids	6 (12)
ICANS (all grades)	1 (2)
Overall mean (SD) infection density per 100 d	0.7 (0.1)
Use of IVIG supplementation	24 (49)

Abbreviations: BCMA, B-cell maturation antigen; bsAb, biospecific antibodies; CRS, cytokine release syndrome; GPRC5D, G-protein coupled receptor family C group 5 member D; FISH, fluorescence in situ hybridization; ICANS, immune effector cell–associated neurotoxicity syndrome; IVIG, intravenous immunoglobulin.

^a^ Data include 42 patients with 49 treatment periods (ie, 7 patients were counted twice). Unless indicated otherwise, data are expressed as No. (%) of patients.

^b^ Data not available in 23 patients.

^c^ Data not available in 7 patients.

^d^ High-risk FISH included t(4;14), t(14;16), del(17p), 1q21.

^e^ Accounted for functional hypogammaglobinemia.

**Figure.**
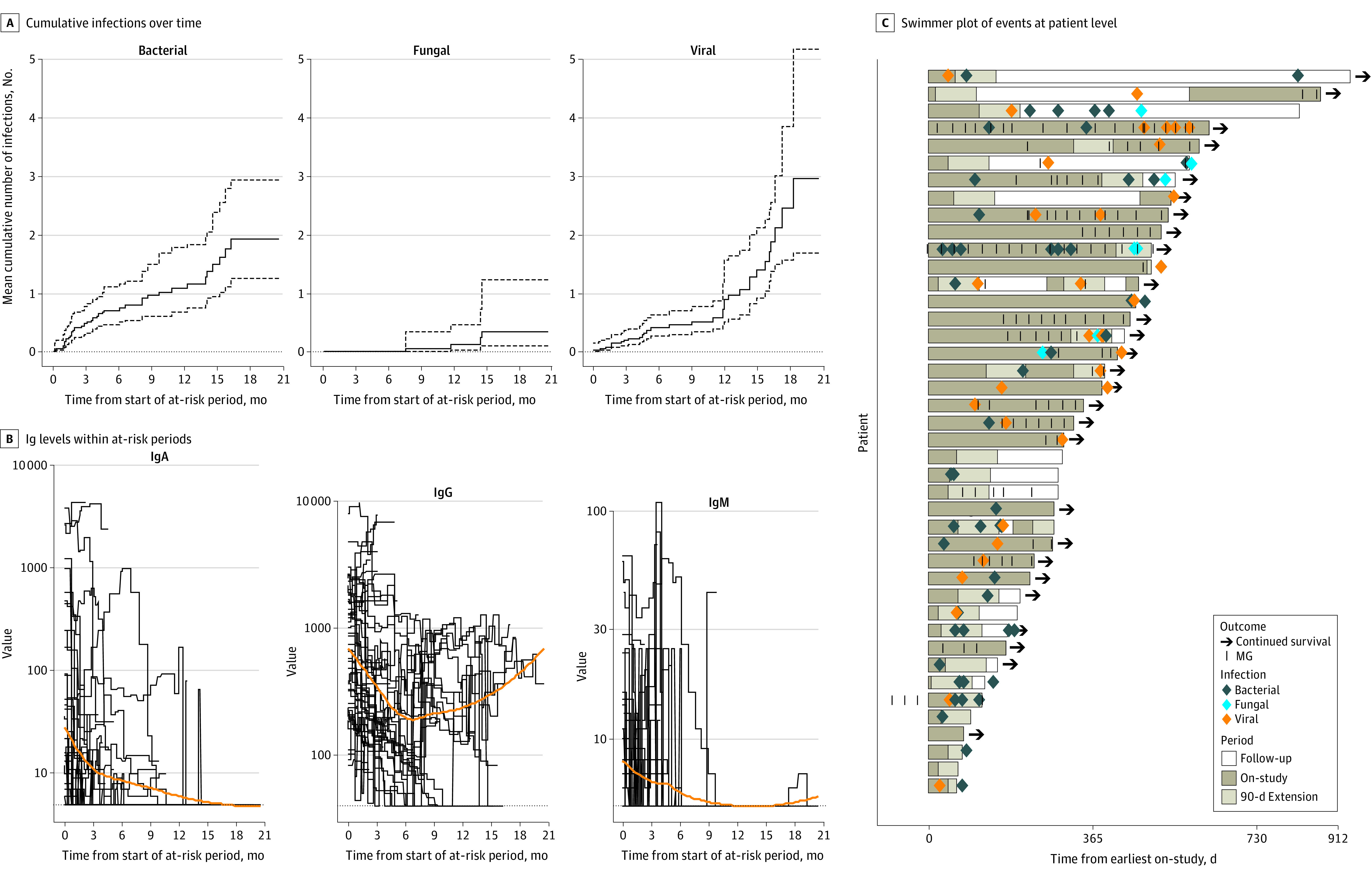
Kinetics of Humoral Deficiency in Relapsed Refractory Multiple Myeloma After Treatment With Bispecific Antibodies A, Mean cumulative number of infections over time. Dotted lines indicate SDs. B, Plot of immunoglobulin levels within at-risk periods with a locally linear smoother curve. The dotted line shows the lower limit of detection. C, Swimmer plot representing each patient 1 time only (n = 42).

The overall cumulative risk of infections increased with increasing duration of therapy with risk at 3 months of 41%; at 6 months, 57%; at 9 months, 64%; at 12 months, 67%; and at 15 months, 70%. The most common infections were bacterial (44 of 81 [54%]), followed by viral (33 of 81 [41%]) and fungal (4 of 81 [5%]) infections. Respiratory tract infections accounted for 41 of 81 infections (51%). Four deaths were attributed to infections, including 2 patients who died within 90 days of treatment (Figure). On multivariate analysis, ongoing bsAb therapy, prior infection, low baseline functional IgG levels, and elevated serum M-spike levels were independently associated with risk of infection. In the current series, 17 patients (40%) received 1 COVID-19 booster, and 2 (5%) received 2 boosters. Although 6 of 16 patients (38%) mounted an anti–spike antibody response to the primary COVID-19 immunization series, this improved to 12 (75%) after booster doses. Four patients had breakthrough COVID-19 infection, 2 after the primary series and 2 after a booster dose.

## Discussion

The findings of this case series highlight the prolonged hypogammaglobulinemia and increased risk of infectious complications with a 70% cumulative infection risk in patients with RRMM treated with bsAb therapy. The risk of infection was associated with the degree of hypogammaglobulinemia and increased with treatment. We also noted an increasing rate of seroconversion with COVID-19 booster vaccination series. Our study is limited by the small sample size and heterogenous patient population treated with differing dose escalations and combinations with other myeloma therapies; nevertheless, our findings underscore the changing continuum of humoral immunodeficiency and infection risk with this growing class of therapeutics in RRMM.

## Notes

Supplement. **eMethods.** Statistical Analysis
