# Supplementary material for: Kinetics of Humoral Immunodeficiency With Bispecific Antibody Therapy in Relapsed Refractory Multiple Myeloma
Source: JAMA Netw Open. 2022 Oct 28;5(10):e2238961. doi: 10.1001/jamanetworkopen.2022.38961 (PMC9617177; doi:10.1001/jamanetworkopen.2022.38961)
Supplement: Supplement. — eMethods. Statistical Analysis [file jamanetwopen-e2238961-s001.pdf]

Supplementary Online Content

Hammons LR, Szabo A, Janardan A, et al. Kinetics of humoral immunodeficiency with bispecific antibody therapy in relapsed refractory multiple myeloma. *JAMA Netw Open*. 2022;5(10):e2238961. doi:10.1001/jamanetworkopen.2022.38961

eMethods. Statistical Analysis

This supplementary material has been provided by the authors to give readers additional information about their work.

© 2022 Hammons LR et al. *JAMA Network Open*.

eMethods. Statistical Analysis

Study variables were summarized by the mean, standard deviation, median, quartiles, and range for continuous variables and frequency and percentage for categorical variables.

Information from 49 treatment occasions of 42 unique patients was imported. Subjects were considered “at-risk” for bispecific antibody (bsAb) associated infection if they were currently on a bsAb study or within 91 days of the last dose. Consecutive periods of at-risk days were combined into “at-risk periods”, and these can combine several on-study to off-study periods, if they are separated by fewer than 91 days. The mean cumulative function estimates the expected average number of infections by any given time point.

The measured IgG/IgA/IgM and serum M levels at a total of 687 occasions in 42 patients were imported. Data from the baseline measurement at the start of the first bsAb treatment was included as day-0 measurements as well.

The following data cleaning steps were performed:
For IgA/G/M and serum M levels:(1) values below the lower limit of detection (eg “<5”) were replaced by the lower limit of detection. (2) “none seen” and “unquantifiable” was set to 0. (3) when IgG and IgA are reported *without* serum M, it was assumed that serum M was too low to be quantifiable and was set to 0.
Functional IgG and IgA levels were computed by subtracting serum M values from the affected immunoglobulin. The value was thresholded at 0.

© 2022 Hammons LR et al. *JAMA Network Open*.
